# Efficacy of Octacalcium Phosphate and Octacalcium Phosphate/Gelatin Composite on the Repair of Critical-Sized Calvarial Defects in Rats

**Published:** 2018-03

**Authors:** Fereydoon Sargolzaei Aval, Mohammad Reza Arab, Narjes Sargolzaei, Fateme Noushadi, Abdolsamad Eteghadi, Asadollah Keykhaei, Foroug Sargolzaei Aval, Azim Hedayat Pour

**Affiliations:** 1 Associate Professor, Cellular and Molecular Research Center, Department of Anatomical Sciences, School of Medicine, Zahedan University of Medical Sciences, Zahedan, Iran; 2 Professor, Cellular and Molecular Research Center, Department of Anatomical Sciences, School of Medicine, Zahedan University of Medical Sciences, Zahedan, Iran; 3 Assistant Professor, Department of Community Medicine, School of Medicine, Zahedan University of Medical Sciences, Zahedan, Iran; 4 Lecturer, Department of Anatomical Sciences, School of Medicine, Zahedan University of Medical Sciences, Zahedan, Iran; 5 General Practitioner, Private Practice, Seattle, Washington, USA; 6 Assistant Professor, Department of Anatomical Sciences, School of Medicine, Tehran University of Medical Sciences, Tehran, Iran

**Keywords:** Octacalcium Phosphate, Gelatin, Bone Regeneration, Parietal Bone, Rats

## Abstract

**Objectives:**

The healing of bone defects in the craniofacial region is an important clinical issue. We aimed to compare the effects of octacalcium phosphate (OCP) and the combination of OCP/gelatin (OCP/Gel) on calvarial bone regeneration in rats.

**Materials and Methods:**

In this study, 72 male Sprague Dawley rats were randomly assigned to the OCP (n=24), OCP/Gel (n=24), and control groups (n=24). Lesions with a diameter of 9 mm were created in the parietal bone and were filled with 9-mg OCP and OCP/Gel disks. In the control group, no substance was implanted in the defect. Sampling was performed on days 10, 14, 21, and 28 after the implantation. After tissue processing, 5-μm sections were prepared and stained by hematoxylin and eosin (H&E) stain. The sections were studied, and the volume fraction of the newly formed bone was assessed by Kruskal-Wallis test at a significance level of 0.05.

**Results:**

In the experimental groups, new bone formation was detected at the margins of the defects 10 days after the implantation. With the progression of the healing process, the newly formed bone covered greater areas of the defects and developed a more mature structure. In the control group, the defects were primarily filled with a dense connective tissue with small islands of new bone. The results of histomorphometric assessments showed that the volume of the newly formed bone in the experimental groups had a significant statistical difference with that in the control group (P<0.001).

**Conclusions:**

The OCP/Gel composite can be useful in the healing process of calvarial bone defects.

## INTRODUCTION

Bone regeneration and healing of defects in the calvaria and maxillofacial bones and after orthopedic surgery are important clinical issues in rehabilitation and regenerative medicine. Bone defects significantly decrease the quality of life of the affected patients [1]. Different clinical solutions have been suggested in order to solve the problems associated with bone damages caused by a lesion, the most important of which are bone grafts [2]. Autogenous grafts are usually the best choice when the defects are small; however, they are inadequate in large defects, and therefore, biologically compatible materials are considered as an alternative [3]. Calcium phosphate (CaP) compounds are one of the most well-known synthetic materials that are able to replace hard tissues [4]. These materials differ according to the amount of phosphate and calcium in their combination and they bear physical and chemical similarities with the natural minerals of bones and teeth [5,6]. Different CaP compounds have various degrees of efficacy in healing bone defects; some act as osteoconductive [7,8] while others, in addition to this property, induce bone formation when implanted in bone defects (osteoinductive) [9,10]. Octacalcium phosphate (OCP), with the chemical formula of Ca_8_H_2_(PO_4_)_6_·5H_2_O, is the most soluble salt among CaP compounds; thus, a lot of attention has been paid to the use of synthetic OCP as a potential loci for bone induction in orthotopic sites with the induction of a significantly higher bone volume compared to the use of other CaP phases such as hydroxyapatite (HA) [11,12] or amorphous carbonated apatite [13]. It has been explained that the biodegradability of OCP is due to its resorption by osteoclast-like multinucleated giant cells (MNGCs) in bone marrow spaces [14,15] after a larger amount of bone deposition compared to HA [15] in addition to its soluble nature in physiological condition. On the other hand, it has been reported that bone formation in the experimentally created large bone defects in the maxillofacial region in animal models (rodent and canine) is regulated by the granule size of CaP compounds [16]. It is likely that a distinct granule size provides distinct intergranular spaces that act as a porous structure encouraging cell migration in a three-dimensional (3D) scaffold [17]. Although OCP has many favorable properties as a bone substitute, it cannot be molded by sintering processes because of its crystal structure and intrinsic properties. In order to overcome these disadvantages and further improve the handling, a combination of OCP and gelatin (OCP/Gel) has been developed [18]. A great deal of effort has been put into forming the bulk of OCP-based materials to be used as an implantable scaffold with a better handling property in clinical situations [19]. Combining OCP with natural polymers not only improves its handling property but also enhances its osteoconductivity [20]. Collagen and gelatin have been examined and proven to enhance the biological properties of OCP in bone regeneration [21,22].

Gelatin, which is a natural material derived from collagen with an almost identical composition, has been widely used as a matrix to obtain biomimetic CaP in bone regeneration and tissue engineering fields [23,24]. In addition, due to its biodegradability, biocompatibility, hydrogel characteristics, availability, and cost-efficiency, gelatin has been increasingly used as the organic moiety in polymer-inorganic hybrids [25].

In an experimental study, Saito et al [26] evaluated the efficacy of OCP/Gel composites in bone regeneration in a rabbit tibial defect model and suggested that the composites had a porous structure of up to 500 μm in diameter and maintained the OCP structure regardless of its content in the gelatin matrix. The OCP/Gel composite with a higher OCP content showed greater bone regeneration and tended to undergo a faster biodegradation in both cortical bone and bone marrow regions. These results suggest that the biodegradation tendency of the composite could be accelerated by increasing the OCP content. This is probably due to the effect of the combination of OCP and gelatin matrix, which results in a higher osteoconductivity [26].

In another study, Saberi et al [27] reported that implantation of OCP/Gel composite was more effective than their separate use in bone regeneration in the defective site, and this combination can be efficiently used for regeneration of mandibular bone defects in rats. In the clinical setting, OCP/ Collagen has been applied in artificially created canine alveolar bone defects and tooth extraction socket models. In these experimental models, OCP/Collagen demonstrated prominent bone regenerative characteristics indicating that OCP/Collagen composite could be a useful bone regenerative material to substitute autogenous bone formation since its implantation could elicit high bone regeneration and active structural reconstitution [28,29]. In a recent study, Kawai et al [30] reported that OCP/Collagen could be used safely for bone regeneration in human models without any infection or allergic reaction. Their results suggested that OCP/Collagen could be a useful bone substitute material to repair large bone defects in humans that might not heal spontaneously [30]. The present study was designed to investigate the effects of OCP and OCP/Gel composite in repairing critical-sized calvarial defects in rats.

## MATERIALS AND METHODS

### Preparation of materials:

Synthetic OCP was prepared by following the methods previously described [31,32]. The sieved granules (particle size of 300–500 μm) of OCP, obtained from dried OCP, were sterilized by heating at 120°C for 2 hours. A previous study showed that such heating does not affect the physical properties such as the crystalline structure or specific surface area of OCP granules [9,33]. Type B gelatin from bovine skin (Sigma-Aldrich, St. Louis, MO, USA) was used in this study. Synthetic OCP/Gel composite was prepared by following the methods previously reported [18,32]. Briefly, gelatin was dissolved in distilled water. The OCP granules were added to the 3% gelatin (Gel) solution and were mixed. The OCP/Gel composite, which contains 44 weight percent (wt%) of OCP granules (OCP44/Gel), was then lyophilized, and the disk was molded (diameter=9 mm, thickness=1 mm). The molded OCP/Gel composite underwent dehydrothermal treatments at 150°C for 24 hours and was then sterilized.

### Implantation procedure:

In this study, 72 adult male Sprague Dawley rats aged 6–8 weeks with an average weight of 120–150 g were used. The rats were obtained from the Animal Research Center of Zahedan University of Medical Sciences and were randomly allocated to two experimental groups (OCP and OCP/Gel) and one control group (n=24 in each group) and were kept in standard conditions with light/dark cycles of equal duration. The principles of the laboratory animal care and the national law were followed. All the procedures were approved by the Ethics Committee for Animal Experiments of Zahedan University of Medical Sciences (IR.ZAUMS.REC.1394–351). General anesthesia was induced by intraperitoneal injection of 60 mg/kg of Ketamine hydrochloride (Ketalar, Trustech Pharma Care, Bayern, Germany) combined with 20 mg/kg of Xylazine (Pantex Holland B.V., Duizel, Netherland) at a ratio of two to one supplemented with ether inhalation. After disinfection of the operation field, skin incisions were made on the parietal bones. The muscle and periosteum of the calvarium were ablated. A full-thickness of standardized critical-sized trephine defect (9 mm in diameter) was made in the calvarium by utilizing a dental handpiece under continuous irrigation with buffered saline. Extreme care was taken to avoid injury to the superior sagittal sinus and dura mater [34]. In our experimental groups, a disk containing OCP/Gel (OCP/Gel group) or OCP granules (OCP group), equal to the size of the created defect (9 mm), was then implanted into the trephine defect, respectively. As a control group, the rats were processed in the same way as in the test groups, except that no implantation was performed after the defects were created.

Afterwards, the periosteum, muscle, and skin were repositioned and sutured by using absorbable 04 sutures (Catgut, Shandong Weigao Group, Kanglida Medical Products Co., Ltd., Heze, China). To prevent infection, 20 mg/kg of Gentamicin (Exir Pharmaceutical Co., Tehran, Iran) was injected intramuscularly for 3 days after the surgery. Based on the study design, sampling was performed during days 10, 14, 21, and 28 after the surgery and implanting the materials.

### Tissue preparation:

In all the groups, general anesthesia was induced by intraperitoneal injection of 60 mg/kg of Ketamine hydrochloride (Ketalar, Trustech Pharma Care, Bayern, Germany), and 10% buffered formalin was perfused through the heart for fixation, and in-situ fixation was immediately done. For a better fixation, the respective area along with a margin of the host bone was resected and stored in 10% buffered formalin at room temperature for one week. The obtained samples were decalcified over four weeks at room temperature by using 10% formic acid (CH_2_O_2_), 2.9% citric acid (C_6_H_8_O_7_), and 1.8% trisodium citrate (Na_3_C_6_H_5_O_7_) [35]. After performing the routine tissue preparation and preparing paraffin blocks, 5-μm sections were serially obtained for histological and histomorphometric studies and were stained by hematoxylin and eosin (H&E) stain. The sections obtained from all the groups on days 10, 14, and 21 were histologically assessed by using light microscopy (Carl Zeiss, Oberkochen, Germany) [36], while histological and histomorphometric assessments were performed on the sections obtained on the 28th postoperative day [18].

### Histomorphometric analysis:

For histomorphometric analysis and determining the volume of the newly formed bone in all the groups, the sections obtained on the 28th postoperative day were used; Six sections were randomly selected from each group (two sections from the surface, two sections from the middle section, and two sections from the deep section of the created defect) and were placed on three slides (three slides and six sections for each sample). Ultimately, 18 slides and 36 sections were selected in each group for histomorphometric evaluation. The sections were stained by H&E stain and were evaluated by using the light microscope equipped with eyepiece graticules at ×40 magnification according to the point counting technique. The mean volume of the newly formed bone was calculated in all the groups and was expressed in percentages [37].

### Statistical analysis:

Histomorphometric data were analyzed by using SPSS software program (version 20, IBM Co. Chicago, IL, USA) and were reported based on the mean, standard deviation (SD), mode, and median. Kruskal-Wallis test was used for comparing the mean volume of the newly formed bone among the groups at a significance level of 0.05.

## RESULTS

### Histological assessments:

**Fig. 1:**
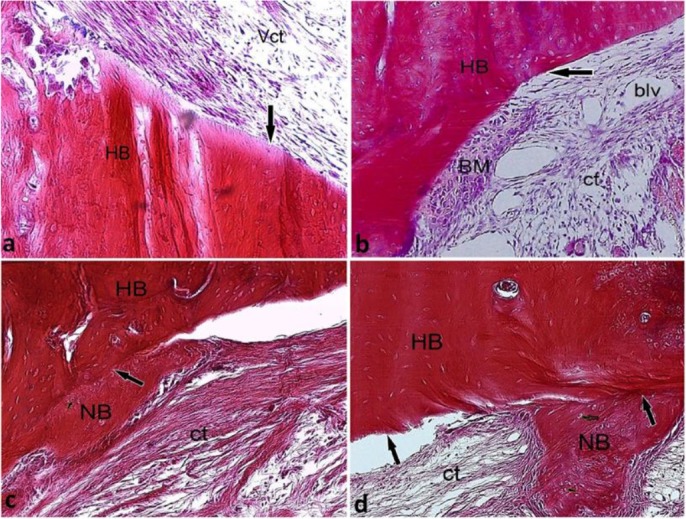
Histological micrograph of the control group on (a) day 10, (b) day 14, (c) day 21, and (d) day 28 post-implantation, H&E staining, ×10 magnification. ct=connective tissue, Vct=vascular connective tissue, HB=host bone, NB=newly formed bone, blv=blood vessel, the defect’s margin (long arrow)

In the control group, the defective area was covered with a highly vascular connective tissue 10 days after the implantation (Fig. 1a).

On the 14th day, the connective tissue filling the defect area developed a better organization pattern, and its adhesion to the defect margins was visible in most parts (Fig. 1b). On the 21st day, the process of bone formation began by the appearance of the eosinophilic bone matrix in areas with the maximum development of organization in the connective tissue (Fig. 1c). At the end of the study period, the newly formed bone was more differentiated and organized (Fig. 1d).

**Fig. 2:**
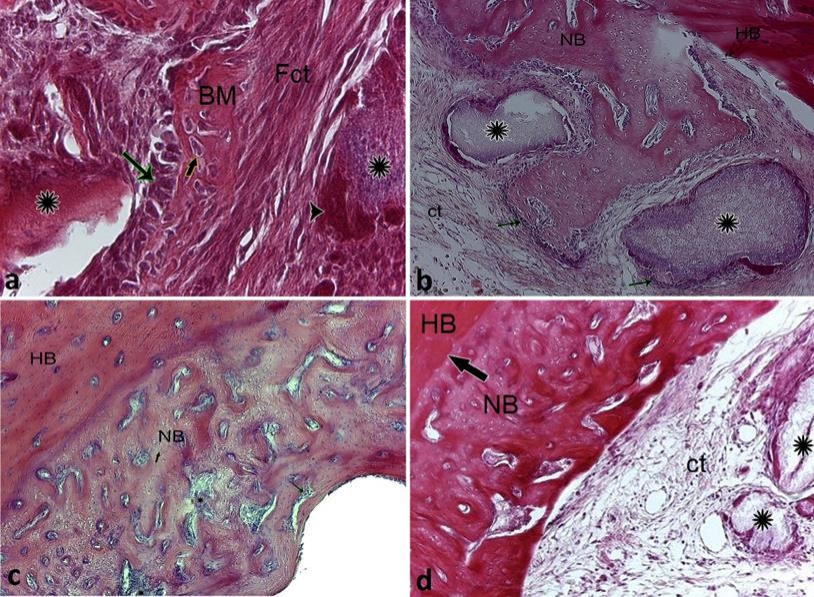
Histological micrograph of the OCP group on (a) day 10, (b) day 14, (c) day 21, and (d) day 28 post-implantation, H&E staining, (a) ×40 magnification, (b, c, and d) ×10 magnification. NB=newly formed bone, implanted OCP particles (*), Fct=fibrous connective tissue, HB=host bone, BM=bone matrix, osteoclast-like cell (arrowhead), epithelioid osteoblasts (thin long arrow), the defect’s margin (thick long arrow)

In the OCP group, on the 10th day, a high density of fibrous elements with differentiating osteoclasts were visible along with epithelioid osteoblasts and the eosinophilic bone matrix (Fig. 2a). On the 14th day after the surgery and implanting the materials, cell differentiation was clearly detectable on the bone surface with the formation of the first Haversian systems and penetration of connective tissue into these osteoblast differentiation areas close to the implanted particles (Fig. 2b). On the 21st day after the implantation, osteon density increased in the newly formed bone, and bone tissue adhesion (the newly formed bone to the host bone) showed a greater development (Fig. 2c). At the end of the study period, in addition to bone formation and the increased volume of the newly formed bone, the implanted particles were gradually absorbed by the MNGCs attached to the OCP particles and were replaced by bone tissue (Fig. 2d)

**Fig. 3:**
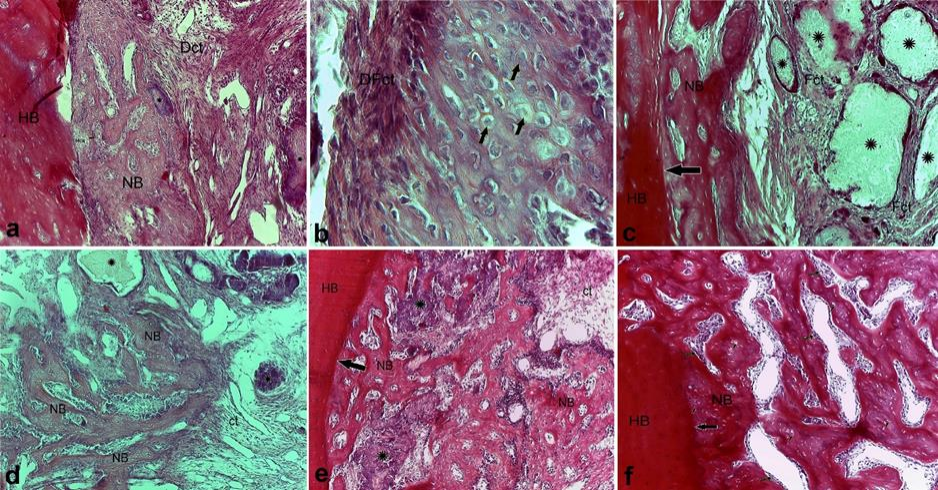
Histological micrograph of the OCP/Gel group on (a) day 10, (b) day 14, (c) day 21, and (d) day 28 post-implantation, H&E staining, (a, c, d, and f) ×10 magnification, (b) ×40 magnification, (e) ×4 magnification. NB=newly formed bone, implanted OCP particles (*), Fct=fibrous connective tissue, Dct=dense connective tissue, hypertrophic chondroblast (short arrow), HB=host bone, the defect’s margin (long arrow)

In the OCP/Gel group, on the 10th day, the newly formed bone could clearly be detected at two areas: at the peripheral area of the defect attached to the host bone, and in the deep and central parts of the defect around the implanted particles (Fig. 3a). Furthermore, at both sites, the induced cells had a spherical shape, hyperchromatic nuclei, and clear cytoplasms indicative of hypertrophic cartilage cells (Fig. 3b). With the progression of the healing process on the 14th day after the implantation, in addition to the growth of the newly formed bone on the margins of the host bone compared to the 10th day (Fig. 3c), the development of new bone started in central areas of the defect and between the implanted particles (Fig. 3d). On the 21st day after the implantation, the new bone attached to the host bone and showed an alternating lamellar-non-lamellar pattern (Fig. 3e). At the end of the study period on the 28th day after the surgery and implanting the materials, a relatively complete pattern of new bone formation, induced by the implanted materials, was observed in all the areas of the defect (Fig. 3f)

### Histomorphometric assessments:

**Fig. 4:**
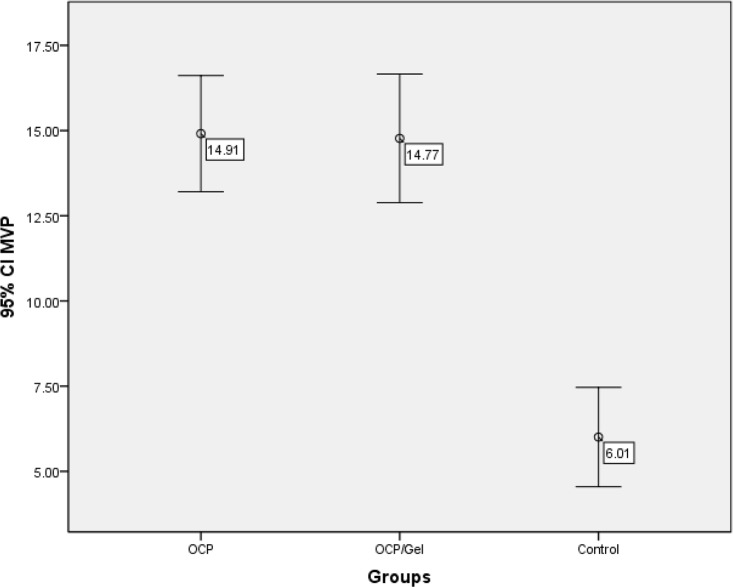
Comparison of the mean volume of the newly formed bone in the experimental and control groups. CI=Confidence interval, MVP=Mean of the volume percent of the new osseous tissue, OCP=Octacalcium Phosphate, OCP/Gel= Octacalcium Phosphate/Gelatin

The volume of the newly formed bone in the experimental and control groups on the 28th day after the surgery and implanting the materials was calculated by using the point counting technique, and the mean value was reported as a volume fraction up to two decimals, the summary of which is presented in Table 1 and Figure 4. Kruskal-Wallis test was used since according to Kolmogorov-Smirnov test, the volume of the newly formed bone in the experimental and control groups did not have a normal distribution. The greatest mean size of the newly formed bone was observed in the OCP group followed by the OCP/Gel group. The control group showed the least amount of the newly formed bone and had significant statistical differences with the experimental groups (P<0.001) indicating the positive effect of the implanted materials on the healing of the parietal bone defects.

**Table 1.** Comparison of the volume of the newly formed bone in the experimental and control groups

**Groups**		**Number of microscopic fields**	**Mean volume percent of the new osseous tissue**	**Standard Deviation**	**Median**	**Mode of the volume percent of the new osseous tissue**	**Minimum**	**Maximum**	**P-value**
**OCP**	OCP/Gel	144	14.77%	11.47	25	25 514%	0	25	0.999
	Control	144	6.01%	8.84	0	0 62.5%	0	25	<0.001

**OCP/Gel**	OCP	144	14.91%	10.34	18	25 38.9%	0	25	0.999
	Control	144	6.01%	8.84	0	0 62.5%	0	25	<0.001

**Control**	OCP	144	14.91%	10.34	18	25 38.9%	0	25	<0.001
	OCP/Gel	144	14.77%	11.47	25	25 51.4%	0	25	<0.001

OCP=Octacalcium Phosphate, OCP/Gel= Octacalcium Phosphate/Gelatin

## DISCUSSION

The effect of synthetic materials, used either alone or combined, on the healing of bone defects has been evaluated in many studies [38,39]. In the present study, the relative capacity of OCP and OCP/Gel to induce osteogenesis after being implanted in the parietal bone defects of rats was evaluated, and the impact of these materials on guiding and advancing the process of bone formation was qualitatively and quantitatively studied by using light microscopy and by histomorphologic and histomorphometric approaches. The data related to the histomorphometric analyses and bone tissue volume calculations were analyzed only on the 28th post-implantation day in order to be able to make a comparison between the experimental groups and the control group. It is worth mentioning that the bone remodeling process is completed by the 28th day, and the new bone is very similar to the primary bone tissue; the only differentiation point would be the reversal lines [18]. The results of the present study showed that in the control group, the bone formation and organization of connective tissue started on the 21st day after the surgery. On the 28th day, the newly formed bone was moredifferentiated and had a greater volume as well as a higher degree of organization compared to the 21st day. The findings of the current study showed that when OCP is implanted in a parietal bone defect, it induces intramembranous bone formation. On the 10th day after implanting the materials, in the central part of the defect adjacent to the implanted particles, a high density of fibrous elements along with differentiating osteoclastic cells were observed, which indicate the start of a new bone formation process by the implanted particles away from the defect margins; this finding has not been reported in previous studies. This result probably reflects the ability of these materials for new bone formation independent from the co-induction influence of the host bone, which is in agreement with the results of a study by Anada et al [40]. On day 21 after the implantation, the materials attached to the bone (the newly formed bone and host bone), and the regular rows of active osteoblasts were visible in the vicinity of the new bone lamellae, which would be another indicator of continuous bone remodeling and bone maturation. Also, the differences in the particle size of the implanted material can reflect the organization process of these particles and the penetration of connective tissue into their depth during 21 days, which is an important finding of the present study. Most of the researchers have reported such a result during the fourth week after implanting the materials [34,41]. At the end of the study period (28 days), it seemed that the volume of the newly formed bone had increased and it was almost indistinguishable from the host bone. This phenomenon was not reported in previous studies on the 28th day after the implantation. The rapid growth of the newly formed bone and early absorption of the OCP implanted particles in this study may be attributable to the positive role of the dura mater in bone cell differentiation [42]. It seems that new bone formation is induced primarily by the host bone in peripheral parts of the defect attached to the host bone and it develops over the center and depth of the defect. In the OCP/Gel group, cartilage tissue masses were detected at the center, depth, and margins of the defect and adjacent to the host bone on the 10th day after the implantation. This phenomenon has not been reported in any of the previous studies. The mechanism of induction of these cells and their reaction in response to the implanted materials are unclear; however, it seems that they can be influenced by the simultaneous interaction between the host bone and implanted materials. Intramembranous formation of new bone tissues was also observed at the margins of the defect attached to the host bone and implanted particles. Another important finding in this group was the penetration of connective tissue into the implanted particles that induced differentiation of osteoblasts and led to the formation of a new bone mass among the implanted particles in the deep parts of the defect. Suzuki et al [43] reported that this phenomenon is caused by the stimulatory effect of OCP on osteoblasts and osteoclasts; these cells and the host osteoblasts can migrate into and through the implanted materials and can cause bone formation in the central parts of the defect and away from the margins of the defect and host bone. This finding is consistent with the results of the present study. At the end of the study, a relatively complete bone formation was observed in all the areas of the defect in addition to the fact that in most cases, the implanted material was completely absorbed. This absorption rate is in contrast with the results of the study by Handa and colleagues [18]. The difference between the results of the mentioned study and the present study may be due to the differences in the percentage of the gelatin used in OCP/Gel composite. The weight percent (wt%) of OCP was 44% in the present study, while the percentage in the research by Handa et al [18] was up to 40%, which led to a slower release of OCP particles in the composite for sixteen weeks after implanting the materials [18].

The histomorphometric findings and comparison of the mean volume of the newly formed bone showed that the volume of the new bone in the experimental groups had a significant statistical difference with that in the control group (P<0.001) indicating the positive effects of the implanted materials, which is consistent with the findings of the study by Suzuki and colleagues [38]. On the other hand, the highest volume of the newly formed bone was related to the OCP group (14.91%) followed by the OCP/Gel group (14.77%) with a slight difference. The volume of the newly formed bone was not statistically different between the two experimental groups (P=0.999); this is probably due to the decreased particle size and a faster absorption of OCP combined with gelatin in the OCP/Gel composite group. The application of OCP, with its osteoconductive and biodegradable nature, in combination with gelatin, with its biodegradability, biocompatibility, and hydrogel characteristics, should be considered where intramembranous bone augmentation is required including rapid bone regeneration and alveolar ridge augmentation in mandibular and maxillary bones.

## CONCLUSION

The present study demonstrated that an OCP/Gel composite containing a high content of OCP granules is able to improve bone formation and defect healing. By adjusting the type of the natural polymer and the OCP form and content, the bone regenerative properties of OCP can be enhanced and biodegradation can be improved, which may be related to the ability of these materials to stimulate osteoclastic activity. Further studies are underway to examine the bone regenerative capacity of OCP/Gel in experimentally created large bone defects in the maxillofacial region.
